# Long non‐coding RNA ZEB1‐AS1 promotes colon adenocarcinoma malignant progression via miR‐455‐3p/PAK2 axis

**DOI:** 10.1111/cpr.12723

**Published:** 2019-12-12

**Authors:** Xin Ni, Yuting Ding, Haitao Yuan, Jinmin Shao, Yan Yan, Rouyu Guo, Wenkang Luan, Min Xu

**Affiliations:** ^1^ Department of Gastroenterology Affiliated Hospital of Jiangsu University Zhenjiang China; ^2^ Department of Rehabilitation Changshu No. 2 People's Hospital (The 5th Clinical Medical College of Yangzhou University) Changshu China; ^3^ Department of General Surgery Affiliated People's Hospital of Jiangsu University Zhenjiang China; ^4^ Department of Liver Disease Zhenjiang Third People's Hospital Zhenjiang China; ^5^ Department of Plastic Surgery Affiliated People's Hospital of Jiangsu University Zhenjiang China

**Keywords:** COAD, growth and metastasis, miR‐455‐3p, PAK2, ZEB1‐AS1

## Abstract

**Objective:**

The long non‐coding RNA zinc finger E‐box‐binding homeobox 1 antisense 1 (ZEB1‐AS1) acts as an oncogenic regulator in many human tumours. In the present study, we identify the role and potential molecular biological mechanisms of ZEB1‐AS1 in colon adenocarcinoma (COAD).

**Methods:**

QRT‐PCR was used to detect the expression of ZEB1‐AS1, miR‐455‐3p and p21‐activated kinases 2 (PAK2) in COAD tissues. CCK8 assay, EdU assay, transwell assay and scratch wound assay were used to explore the biological function of ZEB1‐AS1 in COAD cells. Bioinformatics, luciferase reporter assays and an RNA pull‐down assay were used to demonstrate the mechanism of ZEB1‐AS1. We further explore the role of ZEB1‐AS1 in vivo though xenograft tumour assay.

**Results:**

We found that ZEB1‐AS1 expression was significantly up‐regulated in COAD tissues, and high ZEB1‐AS1 level was correlated with the poor prognosis of COAD patients. MiR‐455‐3p plays an anti‐cancer role in COAD by targeting PAK2. We confirmed that ZEB1‐AS1 promotes PAK2 expression by sponging miR‐455‐3p, thus facilitating COAD cell growth and metastasis.

**Conclusions:**

To sum up, this result illustrates the novel molecular mechanism of ZEB1‐AS1 in COAD and provides a new target for the diagnosis and treatment of COAD patients.

## 1 INTRODUCTION

Colon adenocarcinoma (COAD), a type of colorectal cancer, is one of most common cause of cancer‐related mortality in worldwide.^1^, ^2^ The incidence of COAD in Asian countries has risen sharply in the past decade.^3^ Although many treatments have been used for the management of such cancer, including surgical treatment, radiotherapy and chemotherapy, the 5‐year overall survival rate is still poor.^4^ The main reason for poor prognosis in COAD patients is the lack of reliable biomarkers and therapeutic targets.^5^ Therefore, it is important to find the biomarkers of COAD and explore the potential molecular mechanism of COAD malignant progression.

Long non‐coding RNAs (lncRNAs) play a crucial role in tumorigenesis in different ways, such as chromatin modification, transcription and post‐transcriptional regulation.^6^, ^7^, ^8^ The aberrant expression of lncRNAs can be used as the biomarker in the diagnosis and prognosis of many human tumours.^9^, ^10^ LncRNAs are also involved in the malignant progression of COAD, and some lncRNAs' function in COAD have been confirmed.^11^, ^12^

Long non‐coding RNA zinc finger E‐box‐binding homeobox 1 antisense 1 (ZEB1‐AS1), located in physical contiguity with ZEB1,^13^, ^14^ has been proved to be a cancer‐related lncRNA. ZEB1‐AS1 plays an oncogenic role in many cancers,^15^ such as hepatocellular cancer,^13^ glioma,^16^ gastric cancer^17^ and prostate cancer.^18^ However, the function and molecular biological mechanisms of ZEB1‐AS1 in the malignant progression of COAD remain unclear.

In the present study, we found ZEB1‐AS1 was significantly up‐regulated in COAD tissues. We showed that miR‐455‐3p plays an anti‐cancer role in COAD by targeting p21‐activated kinases 2 (PAK2). PAK2 is a member of the P21‐activated kinases (PAKs) family of serine/threonine kinases.^19^ PAKs, the effectors of the Rho family of small GTPases, participate in a variety of cellular signalling pathways.^20^ Accumulating evidence suggests that overexpression of PAK2 is involved in signalling pathways related to malignant progression of many malignant tumours.^21^, ^22^ We also confirmed the oncogenic effect of PAK2 in COAD. Furthermore, we demonstrated that ZEB1‐AS1 function, as a sponge of miR‐455‐3p, lead to the loss of the inhibitory effect of miR‐455‐3p on PAK2, thus promoting the expression of PAK2. ZEB1‐AS1 promotes the growth and metastasis of COAD via up‐regulating PAK2 level. Thus, ZEB1‐AS1 can act as a new special biomarker or target for the diagnosis and treatment of COAD patients.

## 2 MATERIALS AND METHODS

### 2.1 Human tissues

For this study, we collected 28 paired COAD tissues and adjacent normal tissues (ANT) from COAD patients at The Affiliated Hospital of Jiangsu University. Two pathologists independently diagnosed the pathological features of the tumour tissues. No patients received any neoadjuvant radiotherapy or chemotherapy before operation. This research was endorsed by the Research Ethics Committee of the Affiliated Hospital of Jiangsu University. The Cancer Genome Atlas (TCGA) COAD data are downloaded from the Genome Data Public Data Portal. (https://portal.gdc.cancer.gov/).

### 2.2 Cell lines and cell culture

The primary COAD cell lines (SW480, HT29, LS174T, HCT116 and DLD‐1) and normal human colon histiocytes (CCD‐18Co) were bought from American Type Culture Collection (ATCC). All cells were maintained in Dulbecco's modified Eagle's medium (DMEM; Gibco) containing 10% foetal bovine serum (Thermo Fisher Scientific). COAD cells and normal human colon histiocytes were cultured in a humidified atmosphere of 37°C containing 5% CO_2_.

### 2.3 Oligonucleotides and transfection

miR‐455‐3p mimic, miR‐455‐3p inhibitor and negative control (NC) were bought from GenePharma. The small interference RNAs (siRNAs) for ZEB1‐AS1 and PAK2 was also obtained from GenePharma. The full length of PAK2 was amplified and inserted into a pcDNA3.1 vector (Invitrogen) to construct the PAK2 plasmid. Related oligonucleotides were transfected into COAD cells by using Lipofectamine 3000 (Invitrogen).

### 2.4 Cell proliferation assay

For CCK‐8 (Beyotime) assay, the COAD cells (5000 cells) were seeded in a 96‐well plate, and 100 μL culture media supplemented with 10% CCK8 was added to each well and incubated at different times (12, 24, 36 and 48 hours). The absorbance (450 nm) was measured using microplate reader (Multiscan FC; Thermo Scientific). For EdU assay, EdU imaging kit (Life Technologies) was used to measure the DNA synthesis of COAD cells grown. EdU assay was carried out according to the reagent manufacturer's instructions, and immunostaining and EdU results were visualized using Leica DMI3000B microscope. We counted the positive cells.

### 2.5 Cell invasion and migration assays

For transwell assay, the COAD cells were digested and suspended in serum‐free culture medium. These cells were placed on the top of the Matrigel‐coated chambers (BD Biosciences), and culture medium containing 10% foetal bovine serum was added to the lower chamber as a chemical attractant. After 24 hours, the invading cells were stained and counted. For scratch wound assay, transfected COAD cells were added into a 6‐well plates, and a 200 μL pipette tip was used to form wound gaps. Cells were photographed, and wound widths were recorded at 0 and 48 hours.

### 2.6 Quantitative RT‐PCR

RNA was extracted by using TRIzol (Invitrogen), and reverse transcription was performed using different reverse transcription kits (Applied Biosystems). The PCR amplification reaction was carried out by using StepOnePlus system (Applied Biosystems) according to the set reaction conditions. Special primer of miR‐455‐3p was obtained from RiboBio, and U6 was used for normalization. The primers for ZEB1‐AS1 are as follows: 5ʹ‐CCAGACACCTACACAACTTCC‐3ʹ and 5ʹ‐GTGATCTCTACCCTCTTGCTT‐3ʹ. The relative expression level was calculated using the 2^−△△Ct^.

### 2.7 Western blot

Total proteins were extracted by using RIPA (KenGEN). A BCA Protein Assay Kit (Beyotime) was used to quantify the concentration of protein. Western blotting was carried out according to the previous described.^23^ PAK2 antibody was purchased from Abcam (1:5000) and was used to analyse PAK2 levels, and GAPDH (1:2500; Abcam) was used for normalization.

### 2.8 Luciferase reporter assay

The fragment of PAK2 3′‐UTR and ZEB1‐AS1 containing the binding site of miR‐455‐3p was inserted into the pMIR‐REPORT plasmid. COAD cells were cotransfected with miR‐455‐3p mimic and related reporter plasmids. Mutated plasmid was used as a control. Dual‐Luciferase Reporter Assay System (Promega) was used to measure the luciferase activity.

### 2.9 Isolation of RISC‐associated RNA

Colon adenocarcinoma cells that overexpressed miR‐455‐3p were fixed with 1% formaldehyde. The cells were lysed using NETN buffer and cultured with Dynabeads protein A (Invitrogen) supplemented with IgG or anti‐Pan‐Ago and clone 2A8 antibody (Millipore). Proteinase K digestion was used to release immunoprecipitated RNA. RNA was extracted, purified by ethanol precipitation with glycogen and treated with DNase I.

### 2.10 MS2‐RIP assay

We used maltose‐binding protein (MBP)‐affinity purification to identify miRNAs that associated with ZEB1‐AS1. The MS2‐MBP was expressed and purified from E.coli according to Steitz laboratory method. Three bacteriophage MS2 coat protein‐binding sites were inserted downstream of ZEB1‐AS1 by using Stratagene QuikChange Site Directed Mutagenesis Kit. COAD cells were transfected with MS2‐tagged ZEB1‐AS1 to obtain miRNAs associated with the ZEB1‐AS1. The cells were subjected to RIP analysis after 48 hours. The level of miR‐455‐3p was detected by qRT‐PCR.

### 2.11 RNA pull‐down assay

Biotinylated miR‐455‐3p was bought from GenePharma, and biotinylated mutant and NC were used as controls. Biotinylated miR‐455‐3p, biotinylated mutant and NC were transfected into COAD cells. The cell lysates were incubated with M‐280 streptavidin magnetic beads (Invitrogen).^24^ The bound RNA was extracted, and the ZEB1‐AS1 level was detected by using qRT‐PCR.

### 2.12 Xenograft tumour assay

Fourteen mice (Beijing Laboratory Animal Center) were injected subcutaneously with HT29 cells, and the mice were divided into two groups after the tumours reached 50 mm^3^. ZEB1‐AS1 siRNA or NC was injected into the inner region of the tumour every 4 days for 28 days. The tumour volume (length × width^2^ × 0.5) was measured according to formulas every 4 days. The subcutaneous tumours were removed after 28 days. This research was endorsed by the Research Ethics Committee of the Affiliated Hospital of Jiangsu University. Immunohistochemistry staining of subcutaneous tumours was carried out according to the previously described Ref. 25 and PAK2 antibody (1:150; Abcam) was used.

### 2.13 Statistical analysis

The data are expressed as mean ± standard deviation (SD), and spss13.0 was used for data analysis. *t* test or one‐way ANOVA was used to evaluate the statistical significance. Correlation analysis (spearman) was performed by using matlab. Kaplan‐Meier analysis was used to plot survival curves. *P* < .05 had statistical significance considered to have statistical significance.

## 3 RESULTS

### 3.1 ZEB1‐AS1 was increased in COAD, and high ZEB1‐AS1 level was a risk factor for the prognosis of COAD patients

**Figure 1:**
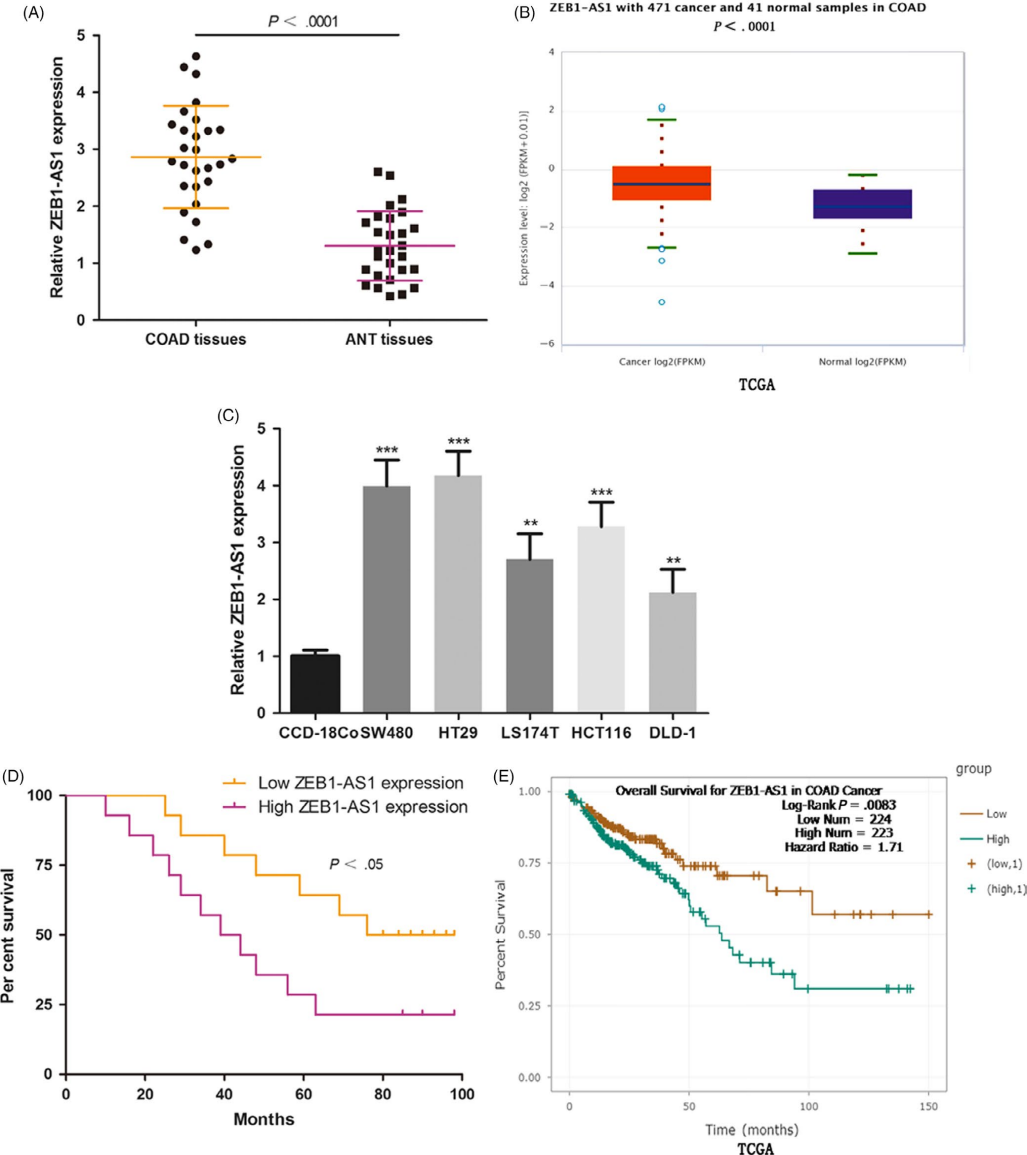
ZEB1‐AS1 was significantly increased in COAD and was found to be a risk factor for the survival of COAD patients. A, The expression of ZEB1‐AS1 was measured in 28 COAD tissues and ANT tissues. B, The expression of ZEB1‐AS1 was analysed in 471 COAD tissues and 41 normal samples by using TCGA database. C, The ZEB1‐AS1 expression profile in primary COAD cell lines (SW480, HT29, LS174T, HCT116 and DLD‐1) and normal human colon histiocytes (CCD‐18Co). D, The overall survival curves of 28 COAD patients. E, The overall survival curves of COAD patients, data from TCGA database. *P < .05, **P < .01, ***P < .001. COAD, colon adenocarcinoma; ZEB1‐AS1, zinc finger E‐box‐binding homeobox 1 antisense 1

We initially analysed the expression of ZEB1‐AS1 in 28 COAD tissues and ANT. ZEB1‐AS1 was significantly increased in COAD tissues compared with ANT (Figure 1A). We discovered the same result by analysing the TCGA‐COAD data set (Figure 1B). Meanwhile, primary COAD cell lines (SW480, HT29, LS174T, HCT116 and DLD‐1) expressed higher ZEB1‐AS1 levels compared with normal human colon histiocytes (CCD‐18Co; Figure 1C). COAD patients with high ZEB1‐AS1 expression (ZEB1‐AS1 expression ratio > median ratio) had a poorer survival (Figure 1D). TCGA‐COAD data set also showed that ZEB1‐AS1 overexpression was correlated with poor survival of COAD patients (Figure 1E). High ZEB1‐AS1 level was closely related to the stage, lymph node metastasis and distant metastasis of COAD, but not to age, family history and sex (Table 1). These results indicated that ZEB1‐AS1 maybe involved in the malignant progression of COAD.

**Table 1:** Correlation between zinc finger E‐box‐binding homeobox 1 antisense 1 (ZEB1‐AS1) level and clinical pathological characteristic (n = 28)

Clinical characteristics	Number	High ZEB1‐AS1 expression	Low ZEB1‐AS1 expression	*P*‐value
Age				
<55	12	5	7	.445
≥55	16	9	7	
Gender				
Male	15	6	9	.256
Female	13	8	5	
Family history				
Yes	5	3	2	.622
No	23	11	12	
Clinical stage				
Ⅰ‐Ⅱ	10	2	8	.018*
Ⅲ‐Ⅳ	18	12	6	
Lymph node metastasis				
Yes	19	12	7	.043*
No	9	2	7	
Distant metastasis				
Yes	16	11	5	.022*
No	12	3	9	

* *P* < .05.

### 3.2 ZEB1‐AS1 promotes the COAD cell proliferation, invasion and migration

**Figure 2:**
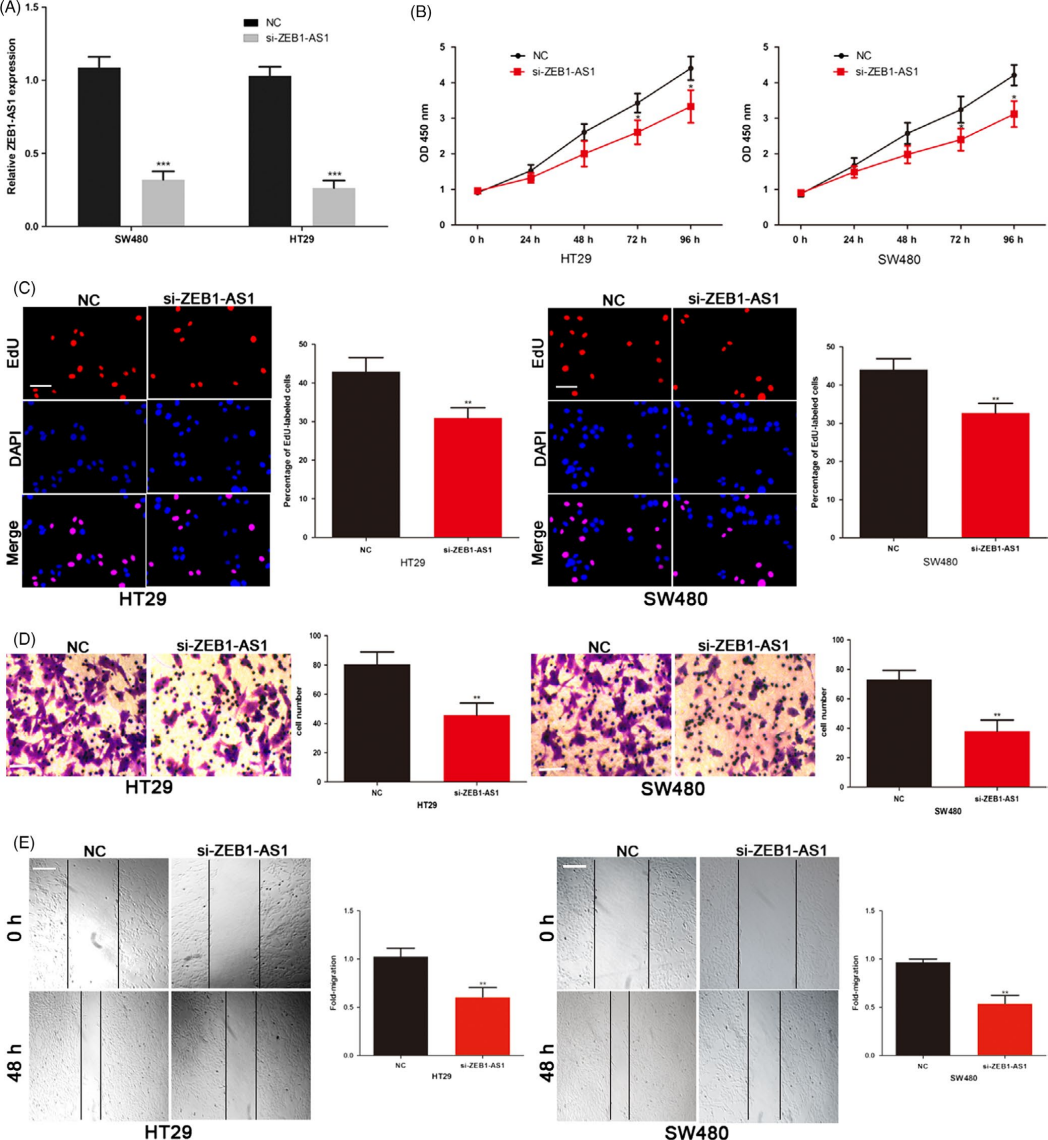
ZEB1‐AS1 promotes the COAD cell proliferation, invasion and migration in vitro. A, Transfection efficiency of ZEB1‐AS1 siRNA was determined by PCR. B, The proliferative ability of SW480 and HT29 cells was determined by CCK8 assay. C, The DNA synthesis of COAD cells grown was measured by EdU assay. Scale bar, 100 μm. D, The effect of ZEB1‐AS1 siRNA on the invasive capacity of COAD cells was assessed by the transwell assay. Scale bar, 50 μm. E, The effect of ZEB1‐AS1 siRNA on the migratory ability of COAD cells was assessed by the scratch wound assay. Scale bar, 200 μm. *P < .05, **P < .01, ***P < .001. COAD, colon adenocarcinoma; ZEB1‐AS1, zinc finger E‐box‐binding homeobox 1 antisense 1

To further explore the biological function of ZEB1‐AS1 on COAD cells, ZEB1‐AS1 siRNA was transfected into SW480 and HT29 cells (Figure 2A). Reduction in ZEB1‐AS1 significantly inhibited the proliferation ability of SW480 and HT29 cells (Figure 2B). Moreover, EdU assay revealed that ZEB1‐AS1 knockdown SW480 and HT29 cells exhibited a marked decrease in the number of EdU‐positive cells (Figure 2C). The invasive and migratory capacities of SW480 and HT29 cells were also repressed in ZEB1‐AS1 siRNA transfected COAD cells (Figure 2D,E). These results indicated that ZEB1‐AS1 can promote the growth and metastasis of COAD.

### 3.3 ZEB1‐AS1 sponged miR‐455‐3p in COAD cells

**Figure 3:**
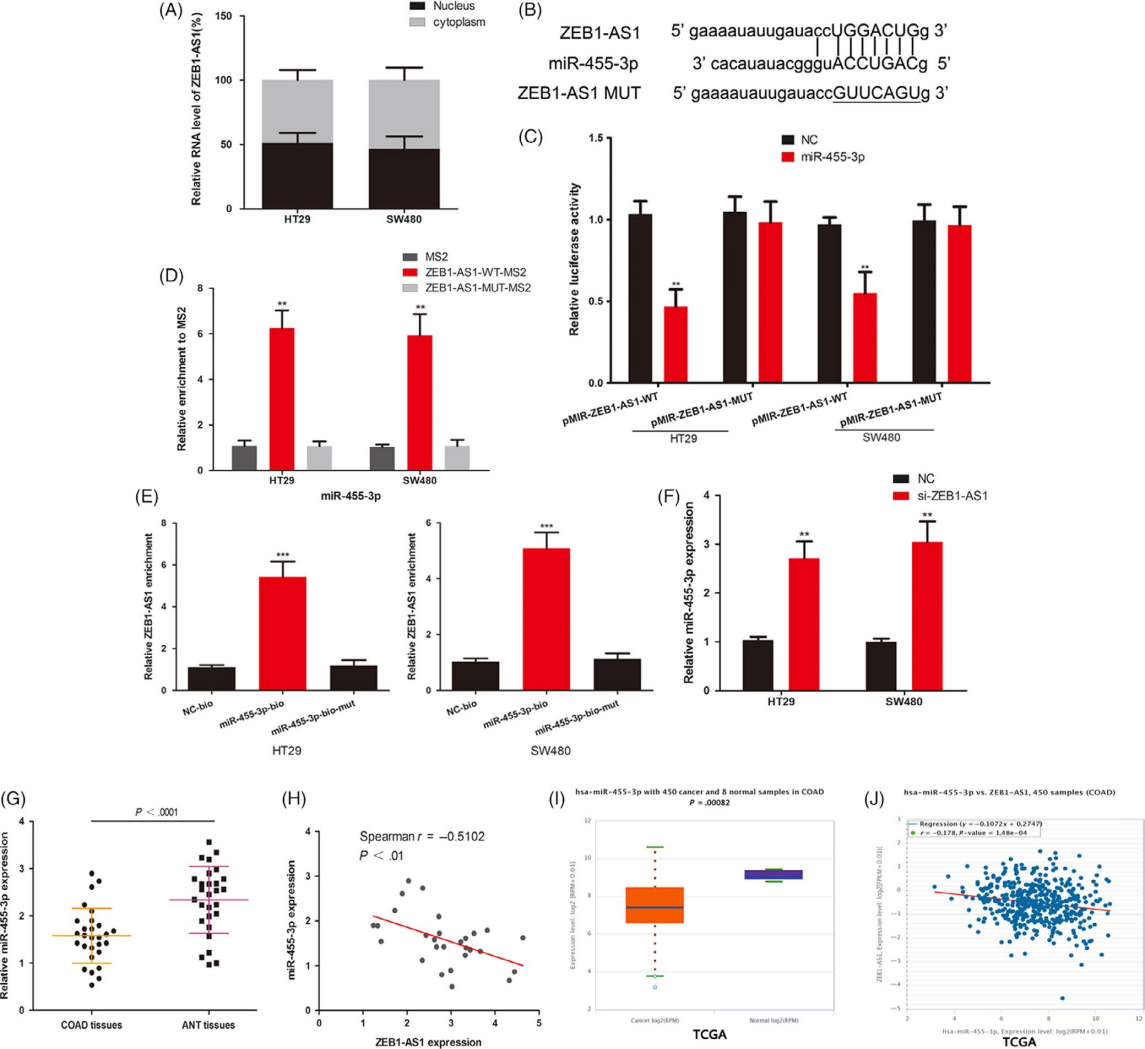
ZEB1‐AS1 sponged miR‐455‐3p in COAD cells. A, RT‐qPCR assays in nuclear and cytoplasmic RNA fractions detected the ZEB1‐AS1 level in cytoplasm and nuclear. B, The binding sites of miR‐455‐3p on the ZEB1‐AS1. C, Luciferase activity of the indicated groups in COAD cells. D, MS2‐RIP followed by miRNA PCR to detect endogenous miR‐455‐3p associated with the MS2‐tagged ZEB1‐AS1 in COAD. E, COAD cells were transfected with biotin‐labelled miR‐455‐3p and assayed by biotin‐based pull‐down after transfection. ZEB1‐AS1 levels were analysed by RT‐qPCR. F, The expression of miR‐455‐3p in COAD cells. G, The expression of miR‐455‐3p was measured in 28 COAD tissues and ANT tissues. H, The correlation of miR‐455‐3p and ZEB1‐AS1 expression in 28 COAD tissues was negative. I, The expression of miR‐455‐3p was analysed in TCGA‐COAD data set. J, TCGA‐COAD data set revealed a negative correlation between miR‐455‐3p and ZEB1‐AS1. *P < .05, **P < .01, ***P < .001. COAD, colon adenocarcinoma; TCGA, The Cancer Genome Atlas; ZEB1‐AS1, zinc finger E‐box‐binding homeobox 1 antisense 1

Some lncRNAs play the role of competing endogenous RNAs (ceRNAs), which can bind to miRNAs to liberate mRNAs targeted by miRNAs.^26^ ZEB1‐AS1 is expressed in both the nucleus and the cytoplasm and also has this effect.^27^, ^28^ RT‐qPCR assays revealed that ZEB1‐AS1 was localized in cytoplasm and nuclear (Figure 3A). Starbase 3.0 (http://starbase.sysu.edu.cn) was used to found potential miRNAs, and miR‐455‐3p may bind to ZEB1‐AS1. We constructed ZEB1‐AS1 luciferase vectors containing the miR‐455‐3p binding sites (wild type and mutated; Figure 3B). MiR‐455‐3p mimics reduced the luciferase activity of wild‐type ZEB1‐AS1 vector in COAD cells (Figure 3C). MS2‐RIP assay was used to further verify the direct interaction between miR‐455‐3p and ZEB1‐AS1 in COAD. The MS2‐tagged wild‐type ZEB1‐AS1 plasmid was enriched for miR‐455‐3p compared with the empty and mutant vectors (Figure 3D). Additionally, we performed an RNA pull‐down assay with biotinylated miR‐455‐3p and found that ZEB1‐AS1 was pulled down by biotinylated miR‐455‐3p in COAD cells (Figure 3E). The miR‐455‐3p level was decreased after transfection of ZEB1‐AS1 siRNA in SW480 and HT29 cells (Figure 3F). Meanwhile, we analysed the level of miR‐455‐3p in 28 pairs of COAD tissues and ANT, and found that miR‐455‐3p was down‐regulated in COAD tissues (Figure 3G). We also found a negative correlation between the expression of miR‐455‐3p and ZEB1‐AS1 in COAD tissues (Figure 3H). TCGA‐COAD database also reveals the same results (Figure 3I,J). All results showed that ZEB1‐AS1 directly binds to miR‐455‐3p in COAD.

### 3.4 ZEB1‐AS1 acts as a ceRNA to promote PAK2 expression in COAD cells

**Figure 4:**
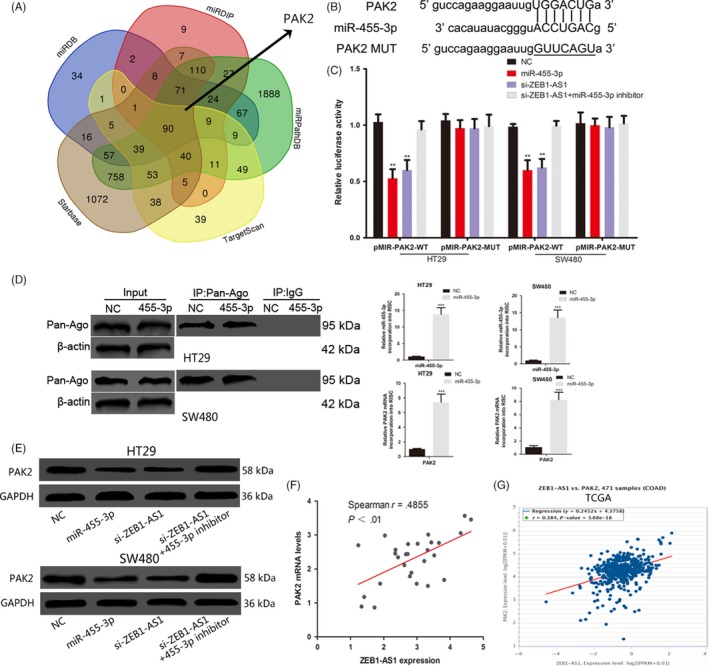
ZEB1‐AS1 acts as a ceRNA to promote PAK2 expression in COAD cells. A, bioinformatics software (TargetScan, miRDIP, Starbase, miRDB and miRPathDB) predict the potential target genes of miR‐455‐3p. B, The binding sites of miR‐455‐3p within the 3′‐UTR of PAK2. C, Luciferase activity of the indicated groups in COAD cells. D, Immunoprecipitation of the Ago2/RISC using the Pan‐Ago2 antibody in COAD cells overexpressing miR‐455‐3p. IgG was used as a negative control, and β‐actin was used as an internal control. PCR analysis of miR‐455‐3p and PAK2 incorporated into RISC in COAD cells overexpressing miR‐455‐3p. E, Western blots identified PAK2 protein expression changes; GAPDH was used as a control. F, The correlation of PAK2 and ZEB1‐AS1 expressions in 28 COAD tissues was positive. G, TCGA‐COAD data set revealed a significant positive correlation between ZEB1‐AS1 and PAK2.*P < .05, **P < .01, ***P < .001. COAD, colon adenocarcinoma; PAK2, p21‐activated kinases 2; TCGA, The Cancer Genome Atlas; ZEB1‐AS1, zinc finger E‐box‐binding homeobox 1 antisense 1

We next applied bioinformatics software (TargetScan, miRDIP, Starbase, miRDB and miRPathDB) to predict the potential target of miR‐455‐3p (Figure 4A). PAK2 was found to have possible targets with miR‐455‐3p, and the 3′‐UTR of PAK2 has the same binding sites that ZEB1‐AS1 combined with miR‐455‐3p (Figure 4B). The luciferase activity of the wild‐type PAK2 vectors was significantly decreased by miR‐455‐3p mimic in COAD cells (Figure 4C). We detected the PAK2 mRNA abundance in the Ago2/RNA‐induced silencing complex (RISC) after overexpression of miR‐455‐3p. Enrichment in the level of miR‐455‐3p and PAK2 that incorporated into RISC was observed in miR‐455‐3p mimic transfected cells by using RNA‐ChIP analysis (Figure 4D). Overexpression of miR‐455‐3p repressed the expression of endogenous PAK2 protein (Figure 4E). These results suggest that PAK2 is the target of miR‐455‐3p in COAD. ZEB1‐AS1 siRNA also inhibited the luciferase activity of the wild‐type PAK2 vectors, and this inhibition can be reversed by miR‐455‐3p inhibitor (Figure 4C). Moreover, ZEB1‐AS1 siRNA repressed the protein levels of PAK2 in COAD cells, and the effect of ZEB1‐AS1 siRNA on PAK2 expression was also attenuated by cotransfection with the miR‐455‐3p inhibitor (Figure 4E). Twenty‐eight COAD tissues collected by us, and TCGA‐COAD data sets showed a positive correlation between ZEB1‐AS1 and PAK2 mRNA level in COAD (Figure 4F,G). To sum up, these results indicated that ZEB1‐AS1 up‐regulated PAK2 expression by competitive binding miR‐455‐3p in COAD.

### 3.5 ZEB1‐AS1 promotes the growth and metastasis of COAD though sponging miR‐455‐3p to up‐regulate PAK2 level

**Figure 5:**
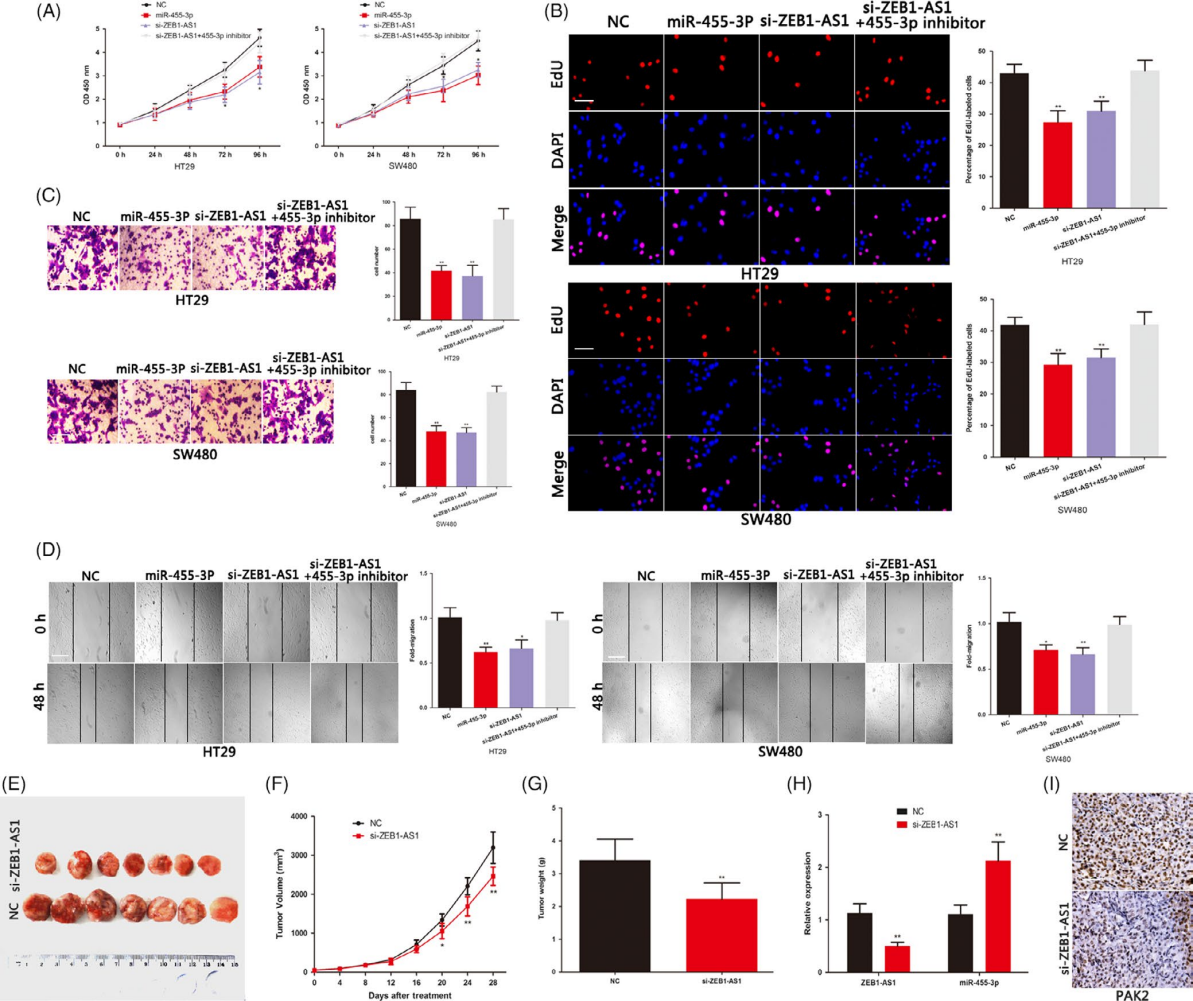
ZEB1‐AS1 promotes the growth and metastasis of COAD by sponging miR‐455‐3p to up‐regulate PAK2 expression. A, The proliferative ability of COAD cells was determined by CCK8 assay. B, The DNA synthesis of COAD cells grown was measured by EdU assay. Scale bar, 100 μm. C, The invasive capacity of COAD cells was assessed by the transwell assay. Scale bar, 50 μm. D, The migratory ability of COAD cells was assessed by the scratch wound assay. Scale bar, 200 μm. E, The excision tumour in nude mice of HT29 xenografts. F, Differences in tumour volume among groups. G, The tumour weight of excised tumour tissues. H, PCR identified miR‐455‐3p and ZEB1‐AS1 expression changes. I, The expression of PAK2 was examined by immunohistochemical staining of sections from the xenograft model. Scale bar, 200 μm.*P < .05, **P < .01, ***P < .001. COAD, colon adenocarcinoma; PAK2, p21‐activated kinases 2; ZEB1‐AS1, zinc finger E‐box‐binding homeobox 1 antisense 1

p21‐activated kinases 2 can promote the proliferation and invasion of many cancer cells. We also confirmed the oncogenic effect of PAK2 in COAD (Figure S1A‐E). We next studied the role of miR‐455‐3p in COAD cells. The miR‐455‐3p mimics inhibited the proliferation, invasive and migratory ability of COAD cells (Figure 5A‐D). The effects of miR‐455‐3p on the COAD cells proliferation, invasion and migration changes were rescued by the PAK2 plasmid (Figure S1A‐E). These indicated that miR‐455‐3p plays an anti‐cancer role by targeting PAK2 in COAD. To study whether ZEB1‐AS1 plays its oncogenic role through sponging miR‐455‐3p, we cotransfected ZEB1‐AS1 siRNA and miR‐455‐3p inhibitors into COAD cells. We found that the inhibitory effect of ZEB1‐AS1 siRNA on growth and metastasis of COAD cells was reversed by miR‐455‐3p inhibitor (Figure 5A‐D). As mentioned earlier, the miR‐455‐3p inhibitor also rescued the effect of ZEB1‐AS1 siRNA on PAK2 level (Figure 4D). We used COAD xenograft model to explore the role of ZEB1‐AS1 in vivo. The excision tumour of nude mice was shown in Figure 5E. As showed in Figure 5F, knockdown group of ZEB1‐AS1 showed significant inhibition of tumour growth between 20 and 28 days. Compared with the control group, the weight of tumours in ZEB1‐AS1 knockdown group was lighter (Figure 5G). The nude mice were sacrificed after 28 days, and the miR‐455‐3p level was increased and that of PAK2 was decreased in the ZEB1‐AS1 knockdown group (Figure 5H,I). Overall, we demonstrated that ZEB1‐AS1 modulates the malignant progress of COAD via sponging miR‐455‐3p to promote PAK2 expression.

## 4 DISCUSSION

Long non‐coding RNA is a non‐coding RNA with a length greater than 200 nucleotides. The abnormal expression of lncRNAs plays a key role in the malignant progression of many malignant tumours.^29^ ZEB1 is a transcription factor that promotes invasion and metastasis of cancer cells.^30^ LncRNA ZEB1‐AS1, located physically adjacent to ZEB1, has been reportedly contributed to various cancers progression.^13^, ^15^ For instance, ZEB1‐AS1 facilitated hepatocellular carcinoma cell growth and motility, and promoted tumour metastasis in vivo.^13^ ZEB1‐AS1 also promotes the proliferation and migration capacities of prostate cancer cells.^18^ It has demonstrated that the high ZEB1‐AS1 level indicates poor prognoses of colorectal cancers.^31^ However, the role and potential mechanism of ZEB1‐AS1 in COAD have not been determined. Here, we found that ZEB1‐AS1 was increased in COAD and conferred a poor prognosis to COAD patients. ZEB1‐AS1 increased the proliferation, invasion and migration ability of COAD cell. Next, we further explored the potential molecular mechanism of ZEB1‐AS1 in COAD.

Numerous studies have shown that certain specific lncRNAs function as ceRNAs, which can sponge miRNAs to regulate the target genes expression of miRNAs.^32^, ^33^ Accumulated evidence indicates that ZEB1‐AS1 also functions as a ceRNA in many human tumours. For instance, ZEB1‐AS1 promotes the tumorigenesis of glioma by regulating the miR‐200c/141‐ZEB1 axis.^34^ ZEB1‐AS1 also facilitates melanoma progression by regulating miR‐1224‐5p.^28^ Here, we showed that ZEB1‐AS1 directly binds to miR‐455‐3p in COAD cells.

miR‐455‐3p plays a role of tumour suppressor in many tumours.^35^, ^36^ In this study, we proved that miR‐455‐3p inhibits the proliferation, invasion and migration of COAD cells via targeting PAK2. PAKs, a member of the STE20 serine/threonine kinases family, play an important role in regulating the changes in actin cytoskeleton structure and cell morphology.^37^ PAK2, a member of PAKs family, is associated with malignant progression of human cancer.^22^ Recent study revealed that PAK2 could mediate tumour cell proliferation, invasion, apoptosis and so on.^38^, ^39^ We confirmed the oncogenic effect of PAK2 in COAD. We discovered that the PAK2 3′‐UTR shares the binding sites of miR‐455‐3p with ZEB1‐AS1. Moreover, we confirmed that ZEB1‐AS1 promotes PAK2 expression by competitive binding miR‐455‐3p in COAD. The inhibitory effect of ZEB1‐AS1 siRNA on COAD cells can be rescued by cotransfecting miR‐455‐3p inhibitor. ZEB1‐AS1 also promotes COAD progression in vivo via miR‐455‐3p/PAK2 axis.

In summary, ZEB1‐AS1 plays a critical role in malignant progression of COAD. ZEB1‐AS1 facilitated the growth and metastasis of COAD cells by competitive binding miR‐455‐3p and promoted PAK2 expression by releasing the PAK2 mRNA transcripts. Understanding the potential molecular mechanism of ZEB1‐AS1 in COAD is important to improve the knowledge of molecular biological basis of COAD development and identify novel special biomarkers or therapeutic target for COAD patient.

## CONFLICT OF INTEREST

The authors report no conflicts of interest in this work.

## AUTHOR CONTRIBUTIONS

WKL and MX conceived and designed the experiments; XN, YTD, HTY, JMS, RYG and YY performed the experiments. WKL, HTY and MX provided the technical support. XN, YTD, MX and HTY analysed and interpreted the data. XN, WKL and YTD wrote the manuscript.

## Data Availability

The data that support the findings of this study are available from the corresponding author upon reasonable request.

## References

[1] Pox CP . Controversies in colorectal cancer screening. Digestion. 2014;89(4):274‐281. 25034478 10.1159/000363287

[2] Schetter AJ , Leung SY , Sohn JJ , et al. MicroRNA expression profiles associated with prognosis and therapeutic outcome in colon adenocarcinoma. JAMA. 2008;299(4):425‐436. 18230780 10.1001/jama.299.4.425 PMC2614237

[3] Kostic AD , Chun E , Robertson L , et al. *Fusobacterium nucleatum* potentiates intestinal tumorigenesis and modulates the tumor‐immune microenvironment. Cell Host Microbe. 2013;14(2):207‐215. 23954159 10.1016/j.chom.2013.07.007 PMC3772512

[4] Hong J , Lu H , Meng X , Ryu JH , Hara Y , Yang CS . Stability, cellular uptake, biotransformation, and efflux of tea polyphenol (‐)‐epigallocatechin‐3‐gallate in HT‐29 human colon adenocarcinoma cells. Cancer Res. 2002;62(24):7241‐7246. 12499265

[5] Tsukuda K , Tanino M , Soga H , Shimizu N , Shimizu K . A novel activating mutation of the K‐ras gene in human primary colon adenocarcinoma. Biochem Biophys Res Commun. 2000;278(3):653‐658. 11095964 10.1006/bbrc.2000.3839

[6] Wang KC , Chang HY . Molecular mechanisms of long noncoding RNAs. Mol Cell. 2011;43(6):904‐914. 21925379 10.1016/j.molcel.2011.08.018 PMC3199020

[7] Mercer TR , Dinger ME , Mattick JS . Long non‐coding RNAs: insights into functions. Nat Rev Genet. 2009;10(3):155‐159. 19188922 10.1038/nrg2521

[8] Cai H , Chen J , He B , Li Q , Li Y , Gao Y . A FOXM1 related long non‐coding RNA contributes to gastric cancer cell migration. Mol Cell Biochem. 2015;406(1‐2):31‐41. 25907137 10.1007/s11010-015-2421-3

[9] Gupta RA , Shah N , Wang KC , et al. Long non‐coding RNA HOTAIR reprograms chromatin state to promote cancer metastasis. Nature. 2010;464(7291):1071‐1076. 20393566 10.1038/nature08975 PMC3049919

[10] Luan W , Zhou Z , Ni X , et al. Long non‐coding RNA H19 promotes glucose metabolism and cell growth in malignant melanoma via miR‐106a‐5p/E2F3 axis. J Cancer Res Clin Oncol. 2018;144(3):531‐542. 29350287 10.1007/s00432-018-2582-z PMC11813268

[11] Zhang Z , Qian W , Wang S , et al. Analysis of lncRNA‐associated ceRNA network reveals potential lncRNA biomarkers in human colon adenocarcinoma. Cell Physiol Biochem. 2018;49(5):1778‐1791. 30231249 10.1159/000493623

[12] Kam Y , Rubinstein A , Naik S , et al. Detection of a long non‐coding RNA (CCAT1) in living cells and human adenocarcinoma of colon tissues using FIT‐PNA molecular beacons. Cancer Lett. 2014;352(1):90‐96. 23416875 10.1016/j.canlet.2013.02.014

[13] Li T , Xie J , Shen C , et al. Upregulation of long noncoding RNA ZEB1‐AS1 promotes tumor metastasis and predicts poor prognosis in hepatocellular carcinoma. Oncogene. 2016;35(12):1575‐1584. 26073087 10.1038/onc.2015.223

[14] Cheng R , Li N , Yang S , Liu L , Han S . Long non‐coding RNA ZEB1‐AS1 promotes cell invasion and epithelial to mesenchymal transition through inducing ZEB1 expression in cervical cancer. Onco Targets Ther. 2018;11:7245‐7253. 30425516 10.2147/OTT.S179937 PMC6203088

[15] Li J , Li Z , Leng K , et al. ZEB1‐AS1: a crucial cancer‐related long non‐coding RNA. Cell Prolif. 2018;51(1):e12423. 10.1111/cpr.12423 PMC6528915 29226522

[16] Lv QL , Hu L , Chen SH , et al. A long noncoding RNA ZEB1‐AS1 promotes tumorigenesis and predicts poor prognosis in glioma. Int J Mol Sci. 2016;17(9):1431. 10.3390/ijms17091431 PMC5037710 27589728

[17] Zhang LL , Zhang LF , Guo XH , Zhang DZ , Yang F , Fan YY . Downregulation of miR‐335‐5p by long noncoding RNA ZEB1‐AS1 in gastric cancer promotes tumor proliferation and invasion. DNA Cell Biol. 2018;37(1):46‐52. 29215918 10.1089/dna.2017.3926

[18] Su W , Xu M , Chen X , et al. Long noncoding RNA ZEB1‐AS1 epigenetically regulates the expressions of ZEB1 and downstream molecules in prostate cancer. Mol Cancer. 2017;16(1):142. 28830551 10.1186/s12943-017-0711-y PMC5568204

[19] Rane CK , Minden A . P21 activated kinases: structure, regulation, and functions. Small GTPases. 2014;5(1):e28003. 24658305 10.4161/sgtp.28003 PMC4160339

[20] Gadepalli R , Kotla S , Heckle MR , Verma SK , Singh NK , Rao GN . Novel role for p21‐activated kinase 2 in thrombin‐induced monocyte migration. J Biol Chem. 2013;288(43):30815‐30831. 24025335 10.1074/jbc.M113.463414 PMC3829397

[21] Gao C , Ma T , Pang L , Xie R . Activation of P21‐activated protein kinase 2 is an independent prognostic predictor for patients with gastric cancer. Diagn Pathol. 2014;9:55. 24621074 10.1186/1746-1596-9-55 PMC3975179

[22] Deng WW , Wu L , Bu LL , et al. PAK2 promotes migration and proliferation of salivary gland adenoid cystic carcinoma. Am J Transl Res. 2016;8(8):3387‐3397. 27648129 PMC5009391

[23] Luan W , Zhou Z , Zhu Y , Xia Y , Wang J , Xu B . miR‐137 inhibits glutamine catabolism and growth of malignant melanoma by targeting glutaminase. Biochem Biophys Res Commun. 2018;495(1):46‐52. 29097210 10.1016/j.bbrc.2017.10.152

[24] Subramanian M , Li XL , Hara T , Lal A . A biochemical approach to identify direct microRNA targets. Methods Mol Biol. 2015;1206:29‐37. 25240884 10.1007/978-1-4939-1369-5_3 PMC6378880

[25] Luan W , Wang Y , Chen X , et al. PKM2 promotes glucose metabolism and cell growth in gliomas through a mechanism involving a let‐7a/c‐Myc/hnRNPA1 feedback loop. Oncotarget. 2015;6(15):13006‐13018. 25948776 10.18632/oncotarget.3514 PMC4536995

[26] Luan W , Li R , Liu L , et al. Long non‐coding RNA HOTAIR acts as a competing endogenous RNA to promote malignant melanoma progression by sponging miR‐152‐3p. Oncotarget. 2017;8(49):85401‐85414. 29156728 10.18632/oncotarget.19910 PMC5689618

[27] Qu R , Chen X , Zhang C . LncRNA ZEB1‐AS1/miR‐409‐3p/ZEB1 feedback loop is involved in the progression of non‐small cell lung cancer. Biochem Biophys Res Commun. 2018;507(1‐4):450‐456. 30448056 10.1016/j.bbrc.2018.11.059

[28] Wang Q , Zhang R , Liu D . Long non‐coding RNA ZEB1‐AS1 indicates poor prognosis and promotes melanoma progression through targeting miR‐1224‐5p. Exp Ther Med. 2019;17(1):857‐862. 30651872 10.3892/etm.2018.7005 PMC6307420

[29] Zhang H , Chen Z , Wang X , Huang Z , He Z , Chen Y . Long non‐coding RNA: a new player in cancer. J Hematol Oncol. 2013;6:37. 23725405 10.1186/1756-8722-6-37 PMC3693878

[30] Larsen JE , Nathan V , Osborne JK , et al. ZEB1 drives epithelial‐to‐mesenchymal transition in lung cancer. J Clin Invest. 2016;126(9):3219‐3235. 27500490 10.1172/JCI76725 PMC5004933

[31] Gong H , Wen H , Zhu X , et al. High expression of long non‐coding RNA ZEB1‐AS1 promotes colorectal cancer cell proliferation partially by suppressing p15 expression. Tumour Biol. 2017;39(6):1010428317705336. 28618933 10.1177/1010428317705336

[32] Tay Y , Rinn J , Pandolfi PP . The multilayered complexity of ceRNA crosstalk and competition. Nature. 2014;505(7483):344‐352. 24429633 10.1038/nature12986 PMC4113481

[33] Liu L , Shi Y , Shi J , et al. The long non‐coding RNA SNHG1 promotes glioma progression by competitively binding to miR‐194 to regulate PHLDA1 expression. Cell Death Dis. 2019;10(6):463. 31189920 10.1038/s41419-019-1698-7 PMC6561933

[34] Meng L , Ma P , Cai R , Guan Q , Wang M , Jin B . Long noncoding RNA ZEB1‐AS1 promotes the tumorigenesis of glioma cancer cells by modulating the miR‐200c/141‐ZEB1 axis. Am J Transl Res. 2018;10(11):3395‐3412. 30662595 PMC6291700

[35] Zheng J , Lin Z , Zhang L , Chen H . MicroRNA‐455‐3p inhibits tumor cell proliferation and induces apoptosis in HCT116 human colon cancer cells. Med Sci Monit. 2016;22:4431‐4437. 27861461 10.12659/MSM.898452 PMC5117242

[36] Liu A , Zhu J , Wu G , et al. Antagonizing miR‐455‐3p inhibits chemoresistance and aggressiveness in esophageal squamous cell carcinoma. Mol Cancer. 2017;16(1):106. 28633632 10.1186/s12943-017-0669-9 PMC5479030

[37] Radu M , Semenova G , Kosoff R , Chernoff J . PAK signalling during the development and progression of cancer. Nat Rev Cancer. 2014;14(1):13‐25. 24505617 10.1038/nrc3645 PMC4115244

[38] Shuang T , Wang M , Shi C , Zhou Y , Wang D . Down‐regulated expression of miR‐134 contributes to paclitaxel resistance in human ovarian cancer cells. FEBS Lett. 2015;589(20 Pt B):3154‐3164. 26363097 10.1016/j.febslet.2015.08.047

[39] Coniglio SJ , Zavarella S , Symons MH . Pak1 and Pak2 mediate tumor cell invasion through distinct signaling mechanisms. Mol Cell Biol. 2008;28(12):4162‐4172. 18411304 10.1128/MCB.01532-07 PMC2423138

