# Supplementary figures and images for: Long non‐coding RNA ZEB1‐AS1 promotes colon adenocarcinoma malignant progression via miR‐455‐3p/PAK2 axis
Source: Cell Prolif. 2019 Dec 12;53(1):e12723. doi: 10.1111/cpr.12723 (PMC6985675; doi:10.1111/cpr.12723)

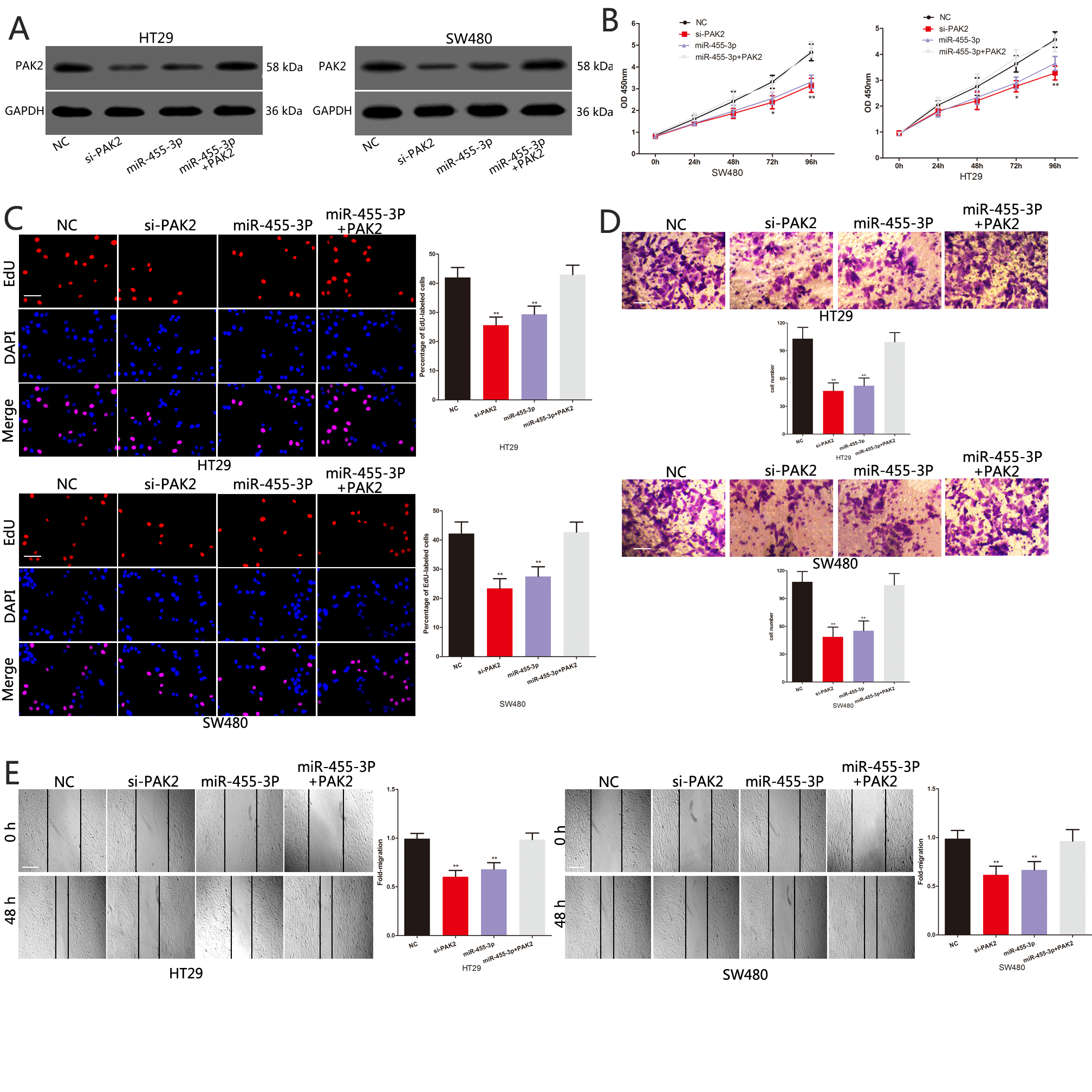

Supplement: Supplementary file 1 [file CPR-53-e12723-s001.tif]
